# The Related Role of Anxiety and Parental Resilience on the Aggressive Tendencies of Preschool Children during the COVID-19 Pandemic

**DOI:** 10.3390/children11060661

**Published:** 2024-05-29

**Authors:** Evrim Durgut Şakrucu, Haktan Demircioğlu

**Affiliations:** 1 Ankara Training and Research Hospital, 06230 Ankara, Türkiye; 2 Department of Child Development, Hacettepe University, 06800 Ankara, Türkiye; hdemircioglu@hacettepe.edu.tr

**Keywords:** preschool children, anxiety, aggressive tendencies, parental resilience, COVID-19 pandemic

## Abstract

In this study, we aimed to determine the related role of anxiety and parental resilience on the aggressive tendencies of preschool children during the COVID-19 pandemic. The study sample comprised a total of 293 parents with children aged 4–6 years. Relationships between Preschool Anxiety Scale, Aggression Orientation Scale, and Brief Resilience Scale total and subscale scores were evaluated using Pearson and Spearman correlation analysis. The results of this study show that there is a positive relationship between children’s aggression tendencies and anxiety levels. We observed weak to moderate correlations between parents’ resilience scores and the children’s aggression and anxiety scores. Although linear regression analysis indicated no significant effect of parental resilience on children’s aggressive tendencies, anxiety levels may be related. In addition, study results showed that the physical aggression tendencies of children differed according to their age and the number of children in the family, albeit at a low level. Further studies are needed to identify factors associated with aggression in preschool children.

## 1. Introduction

Declared a pandemic by the World Health Organization in March 2020, COVID-19 has caused not only health problems, but also many economic, social–emotional, and societal problems. The prolongation of the pandemic led to many changes in the lifestyles of children as well as adults. Moreover, children are more vulnerable to traumatic events such as natural disasters and epidemics because, unlike adults, they do not have the experience and skills to access the resources necessary to meet their developmental, social–emotional, and health needs [1]. Therefore, listening to what children believe about COVID-19 transmission is essential; providing children with an accurate explanation that is meaningful to them will ensure that they do not feel unnecessarily frightened or guilty [2]. Stress such as boredom, lack of information about the situation, being separated from friends and teachers, fear of infection, lack of personal space at home, and family financial losses can have lasting effects on children and adolescents [3]. In a study, it was stated that during the COVID-19 pandemic, a significant increase in fear and anxiety, having problems with siblings, restlessness, aggression behaviors, and psychological-based physical illness complaints were observed in school-aged children [4].

One of these potential adverse effects is aggressive behavior, which is the most common reason children and adolescents are referred for psychiatric assessment and care [5].

Childhood aggression is also associated with maladjustment, depression, emotional disorders, victimization, and poor peer relationships [6]. Many children begin displaying physical aggression at the age of one or two years in response to frustration or as a means of achieving their goal. The first aggressive behavior seen with peers is forcefully taking toys from other children, which is soon followed by hitting. Physical aggression tends to increase between 30–42 months of age and then decreases as children gain the ability to regulate their attention and emotions, control their impulses, and use verbal communication to resolve conflicts and express their needs [7]. Another type of aggressive behavior seen in this period is relational aggression. Relationally aggressive behavior in general is quite damaging to peer relationships and the child being exposed to it, albeit inconspicuously. Relational aggression refers to peer exclusion, spreading secrets or falsehoods, and other behaviors that may manifest both verbally and nonverbally (e.g., avoiding or ignoring certain peers [8]). In the preschool period, relational aggression is typically observed as behaviors such as putting a hand on a chair to keep another child from sitting there or telling another child, “I don’t want you to be my friend” [9]. Also, there is a positive relationship between showing aggressive behavior towards peers and being exposed to aggressive behavior by peers in preschool children. Children who are exposed to these behaviors may also show violence to other children [10].

The preschool period is developmentally important in terms of learning appropriate social behaviors [11]. Aggressive behaviors will negatively affect a child’s relationships with peers and make social acceptance difficult. Aggressive behaviors that emerge during this period can turn into lifelong problem behaviors. Understanding the development of aggressive behaviors in the preschool period is also very important because it is a strong predictor of violent or nonviolent negative behaviors in adolescence. Some studies indicate that aggressive behaviors exhibited in the preschool period predict aggressive and delinquent behaviors in adolescence [12]. In this context, being able to determine aggressive tendencies in the preschool period will also contribute to prevention and intervention studies related to problems that arise later in life.

Another condition that may be seen in early childhood in association with the COVID-19 pandemic is anxiety disorder. Anxiety has a broad impact on children’s social–emotional development. It is often associated with insomnia, physical problems, and academic difficulty [13]. A study in China found that children ages 3 to 18 experienced high levels of anxiety during the COVID-19 pandemic, and reading, digital games, and physical activities were found to be effective in coping with this situation [14]. Anxiety symptoms in early childhood are risk factors for anxiety disorders in adolescence and adulthood [15]. Because, anxiety is not just a temporary childhood problem, it is a condition that negatively affects children’s psychosocial development in both the short and long term [16]. It is also stated that parents being overly anxious, intrusive, controlling, and less compassionate is strongly associated with shyness and anxiety in their children [17]. One study showed that pregnancy-related anxiety experienced by mothers at 24 and 34 weeks of gestation was related to the emotional and behavioral difficulties experienced by their children at the age of 4 years [18]. Hudson, Dodd, and Bovopoulos (2011) determined, in their study of preschool children, that both a child’s temperament and family environment influence their anxiety level. They stated that children who grow up in a negative family environment are more anxious, and children’s anxiety is especially related to the mother. They stated that the reason for this is that mothers tend to be more negative when talking and interacting with their children [19].

In the COVID-19 pandemic period, behavior changes and comfort changes within the scope of family relations, as well as individuals, brought about psychosocial difficulties and relationships in the roles of family members [20]. In this context, parents’ psychological resilience is crucial in terms of their communication with the child. Individuals’ reactions and coping strategies may differ in the face of negative experiences. Some individuals may have problems such as anxiety and depression in response to stressful and traumatic events, and these problems may persist in the long term. Others can overcome these negative emotions and return to normal within a short time. The power of individuals to recover from adversity quickly is described as psychological resilience [21]. Many researchers have proposed different ideas about resilience. For example, resilience is thought to be a trait that develops through exposure to stress and difficulties [22], but some researchers consider it a dynamic process rather than a static trait [23]. Resilience has also been defined as the ability to successfully manage distress and stress [24]. In the literature, there are studies examining the effects of the COVID-19 pandemic on children. However, this study is important in terms of investigating the effects of children’s anxiety states and their parents’ resilience on the aggression tendencies of preschool children during the pandemic.

In this study, we aimed to determine the related role of anxiety and parental resilience on the aggressive tendencies of preschool children during the COVID-19 pandemic period. We sought answers to the following research questions:

1. Is there a relationship between children’s aggressive tendencies and anxiety levels?
2. Is there a relationship between children’s aggressive tendencies and parents’ resilience levels? Do parental anxiety and resilience levels predict children’s aggressive tendencies?

## 2. Method

### 2.1. Study Design and Sample

This research was designed according to the relational model, one of the quantitative research methods. Relational research is a nonexperimental research study designed to explain whether and what kind of relationship exists between two or more variables and to make predictions about variables [25,26]. The study sample comprised a total of 293 parents with children aged 4–6 years.

### 2.2. Data Collection Tools

The children’s aggressive tendencies were assessed using the Aggression Orientation Scale for Children Aged 36 to 72 Months. This instrument was developed by Kaynak, Kan, and Kurtulmuş in 2016 and consists of 27 items in 4 subscales: physical aggression towards others, relational aggression towards others, self-aggression, and aggression towards objects. The scale was found to have acceptable content and construct validity, and criterion validity was also examined by using the Preschool Social Behavior Scale–Teacher Form, which was adapted into Turkish [27]. The scale also showed good reliability, with a Cronbach’s alpha coefficient of 0.957 for the whole scale and subscale Cronbach’s alpha coefficients of 0.946 for physical aggression towards others, 0.945 for relational aggression towards others, 0.853 for self-aggression, and 0.920 for aggression towards things/objects. Thus, the Aggression Orientation Scale for Children Aged 36 to 72 Months was concluded to be valid and reliable [28]. Each item in the scale has a value from 1 to 7. These points are added together to reach the total score. Additionally, there are no reverse-coded scores on the scale. Sub-dimensions are reached by summing the relevant items. The sum of these sub-dimensions gives the aggression score. Also, the original instrument was used in the study.

The children’s anxiety tendencies were assessed using the Revised Preschool Anxiety Scale. Developed by Edwards, Rapee, Kennedy, and Spence [29], the Revised Preschool Anxiety Scale was adapted into Turkish by Güler in 2016. This scale includes a total of 30 items in 4 subscales: social anxiety, generalized anxiety, separation anxiety, and specific fears. All item-total correlations were higher than 0.30, indicating sufficient internal consistency, and item discrimination values were between 5.40 and 14.90 according to the lower and upper groups, showing a high discrimination level. In reliability analyses, Cronbach’s alpha was found to be 0.90 and McDonald’s omega value was found to be 0.92. Considering that the reliability level for the measurement tools should be 0.70 or higher, the Revised Preschool Anxiety Scale was regarded as having high reliability [30]. There are no reverse-coded scores in the scale. Sub-dimensions are reached by collecting relevant items. Additionally, the sum of these sub-dimensions gives the anxiety score. Each item in the scale has a value from 1 to 5: “not true at all” is 1 point; “most of the time true” is 5 point. A high score indicates high anxiety. The Turkish version of the scale was used in our study.

The family’s resilience tendencies were assessed using the Brief Resilience Scale. This instrument was developed by Smith et al. to measure individuals’ resilience levels [31]. It was adapted into Turkish by Doğan in 2015. This scale has no subdimensions and consists of 6 items in total. Analysis of its criterion validity using the Oxford Happiness Questionnaire–Short Form (OHQ-SF), Ego-Resiliency Scale (ERS), and Connor-Davidson Resilience Scale demonstrated significant positive correlations with ERS and OHQ-SF scores [19]. Items 2, 4, and 6 are reverse-coded. Other items were coded directly and the total score was calculated.

### 2.3. Data Collection Process

Ethics committee approval was obtained from the Hacettepe University Ethics Committee (11.12.2020/E-35853172-000-00001359343). Due to the pandemic, data collection was carried out online via Google Forms. The forms were sent to 14 preschools in Ankara, who forwarded them to the parents of enrolled children. Families filled out the informed consent form indicating their voluntary participation in the study. Of the parents participating in the study, 92.6% were mothers and 7.4% were fathers. Considering the age of the mothers participating in the study, 50.2% of them were 31–35 years old, 22.3% were 30 and under, and 20.8% were 36–40 years old. The age of fathers was 38.2% 31–35 years old, 34.3% 36–40 years old, 20.5% (n:50) 41 and over, and 7.1% (n:20) 30 and below. Considering the gender of the children, 50.9% were female and 49.1% were male.

### 2.4. Data Analysis

Relationships between Preschool Anxiety Scale, Aggression Orientation Scale, and Brief Resilience Scale total and subscale scores were evaluated using Pearson and Spearman correlation analysis. Multiple regression analysis was performed to determine whether children’s anxiety and parental resilience are predictors of the aggression in children.

## 3. Results

In our analysis of the relationship between the children’s aggression tendencies and anxiety levels, there were statistically significant but weak positive correlations between Preschool Anxiety Scale total and subscale scores and Aggression Orientation Scale total score and all subscale scores, except in the self-aggression subscale (Table 1). Whether the obtained values were significant or not, a significance level of 0.05 was used as a criterion for interpretation.

There were also significant, weak to moderate negative correlations between Brief Resilience Scale scores and all Aggression Orientation Scale and Preschool Anxiety Scale scores, except for the self-aggression subscale (Table 2).

In correlation analysis between the children’s aggression and anxiety scores and their age and number of children in the household, physical aggression subscale and total aggression scores showed weak negative correlation with age and weak positive correlation with number of children in the family. There were no other significant associations among these variables (Table 3).

The results of the multiple linear regression analysis between anxiety, parental resilience, and the children’s aggressive tendencies are presented in Table 4. Regression analysis is a method that seeks to predict a dependent variable based on data pertaining to independent variables. The Durbin–Watson value was found to be 1.95, indicating no autocorrelation between the error terms, and the variance amplification factor (VAF) of 1 demonstrated that there was no multicollinearity problem. Preschool Anxiety Scale scores were significant in the model (adjusted *R*^2^ = 0.06, *p* < 0.05), whereas Brief Resilience Scale scores were excluded from *p* > 0.05. The adjusted *R*^2^ value indicated that Preschool Anxiety Scale scores explained 6% of the variance in Aggression Orientation Scale scores. Based on the standardized regression coefficient (β), we determined that a 1-standard-deviation change in Preschool Anxiety Scale score resulted in a 0.26-point change in Aggression Orientation Scale score (*p* < 0.05).

## 4. Discussion and Conclusions

The current study examined the related role of anxiety and parental resilience in the aggressive tendencies of preschool children during the COVID-19 pandemic period. We found that there is a positive relationship between children’s aggression tendencies and anxiety levels. On the other hand, we observed weak to moderate correlations between parents’ resilience scores and the children’s aggression and anxiety scores. Children are more vulnerable than adults to traumatic events such as natural disasters and epidemics. For this reason, during the COVID-19 epidemic, a significant increase in fear and anxiety, restlessness, aggressive behavior, and psychologically based physical illness complaints was observed in children. In this context, we think that the findings of the study will contribute to the field.

Anxiety is a potential pandemic-related problem with serious implications for children’s social–emotional development and mental health in adolescence and adulthood. The results of our study suggest there may be a positive relationship between children’s aggressive tendencies and their anxiety levels (Table 1). This may be because preschool children experience psychosocial restlessness due to their inability to make sense of their anxiety, which then turns into uncontrolled anger.

Our results in the present study support the relevant literature. One study indicated that anxiety in childhood is a risk factor for prospective aggression in children, together with increasing family conflicts [32]. The results of epidemiological studies have shown that the early onset of aggressive behaviors was associated with higher frequency of anxiety and social phobia later in life [33]. The COVID-19 pandemic, which has been ongoing since 2019, caused many long-lasting changes in children’s lives. One of the negative consequences of these changes is aggressive behavior [5]. Aggressive behaviors seen in childhood increase the risk of developing problems such as physical violence, guilt, and relationship difficulties in later life [34]. Among the reasons for negative behaviors such as aggression, stubbornness, and crying seen in children during the COVID-19 pandemic process, their inability to spend their energy and experiencing space restrictions can be counted. In a one-year longitudinal study conducted with third-grade elementary school students, it was determined that relational aggression was a risk factor for future physical aggression and anxiety [35]. Aggression and anxiety are also reported to be important indicators for the mental health of adolescents. A study conducted with adolescents showed that being at high risk for anxiety was significantly associated with total aggression scores, and that relational aggression was more associated with anxiety than physical aggression [36].

Although it is seen that there is a relationship between aggressive behaviors and anxiety in children and that most children who display aggressive behaviors also show anxiety symptoms, this situation is often overlooked. In fact, anxiety should be included among the primary targets in intervention programs for aggressive behaviors in childhood [37]. Based on the similarities between our findings and the results of other studies, it can be said that unexplained anxiety strengthens the potential for aggressive behavior in childhood and adolescence. In this context, it is very important to control the anxiety experienced in response to situations in the early period and transform the irrational thoughts that cause anxiety into realistic ones. Although there is an increase in the severity of anxiety and aggression in the COVID-19 process, it has been reported that increasing anxiety intensity also increases the severity of aggression [38].

Research findings point to a relationship between parents’ levels of psychological resilience and children’s levels of aggression and anxiety. In the present study, we observed weak to moderate correlations between the parents’ resilience scores and children’s aggression and anxiety scores (Table 2). Resilience, which is defined as the ability to manage boredom and stress well, is very important as it expresses the power of parents to bounce back quickly from negative moods caused by adverse experiences, such as the COVID-19 pandemic. It is undeniable that parental attitudes and behaviors are important in a child’s development and impact the behaviors the child will acquire. For this reason, it is important to evaluate the role of parental resilience in the parent–child relationship during the pandemic. Parental attitudes, which differ in each family, play a major role in the formation of personality traits as well as the behavior of the child. Children who grow up in families with positive, balanced, and democratic attitudes have more positive personality traits and behavior patterns than those in families with negative attitudes [39,40]. In addition, studies have found that an authoritarian parental attitude increases the relational aggressive behaviors seen in children [41]. As a result of negative parental attitudes and insufficient support for their social–emotional development in the preschool period, children may show problem behaviors such as aggression or bullying [42]. Children who engage in aggressive behavior may experience communication problems with those around them, their self-perception may be negatively affected, and they may experience mental problems in the future [43]. On the other hand, especially in the COVID-19 pandemic, when children were kept home and the burden of childcare and working were placed primarily on parents, it is likely that child aggression in the home influenced parental resilience and ability to cope with those circumstances.

Our study results showed that the physical aggression tendencies of children differed according to their age and the number of children in the family, albeit at a low level (see Table 3). Consistent with our findings, previous studies have shown that the presence of siblings is a factor in physical aggression levels in early childhood. Especially, children with younger siblings show more aggressive behaviors [44]. It was also reported that children with one or more siblings had higher aggression levels than only children [45]. On the other hand, there are studies in the literature suggesting that only children exhibit more problem behaviors than children with siblings [42] or that sibling number is not related to aggressive behavior [46]. Dizman reported that the aggression levels of children changed according to birth order and this level was lower in first-born children [45]. This was attributed to the fact that first-born children are given more responsibility than other children and communicate more with their parents, resulting in less aggressive behavior compared to later-born children.

In this study, parents’ resilience scale scores negatively correlated at a low level with children’s social anxiety, separation anxiety, specific fears, physical aggression, and total aggression scores, and at a moderate level with their generalized anxiety and total preschool anxiety scores (see Table 2). Although our linear regression model indicated no significant effect of parental resilience on children’s aggressive tendencies, anxiety levels may be related. Preschool Anxiety Scale score explained 6% of variance in Aggression Orientation Scale score, with a change of 1 standard deviation in anxiety score associated with an increase of 0.26 points in aggression orientation scores (see Table 4). This suggests that despite weak to moderate correlation between parental resilience and children’s aggression and anxiety, only the children’s anxiety scores predict their tendency toward aggression. This may stem from parents’ inability to teach children how to manage their anxiety. On the other hand, although we concluded that only the child’s anxiety is associated with children’s aggressive tendencies, it may be that parental resilience, which was found to be associated with anxiety, has an indirect rather than a direct effect on their aggression orientation. Since aggressive behaviors make it difficult for the child to develop self-esteem and self-control, exhibiting these behaviors in early childhood has negative consequences for both the child and their environment [47]. In this respect, it is very important to determine all variables that affect these behaviors in order to prevent the negative effects of aggression and provide necessary interventions. Also, the preschool period is very important in a child’s development, forming the basis for the later stages of life. Therefore, it is essential to spend this period in a healthy, conscious, and meaningful way [48].

Disease outbreaks, such as the COVID-19 pandemic, affect not only the physical health of individuals but also the psychological health of the uninfected population [49]. It is now a known fact that stressful events are powerful negative environmental factors that can predispose individuals to psychiatric disorders, especially depression [50]. Although children’s perception and understanding of events continue to develop throughout childhood and adolescence, studies indicate that even children under the age of 2 are aware of the changes around them [51]. For this reason, the development of children who are more vulnerable than adults and who face a crisis situation such as a pandemic but cannot benefit from mental health services may be negatively affected [52]. In the early days of the epidemic, more attention was naturally given to the physical consequences of the virus, and its effects on mental health were not taken into consideration. However, even if the pandemic is over and we return to our normal lives, its psychological effects will likely last for months or even years. For this reason, we think that our study on aggressive behavior, one of the problem behaviors seen in children during the pandemic period, will contribute to the literature in terms of child mental health.

**Figure 1:**
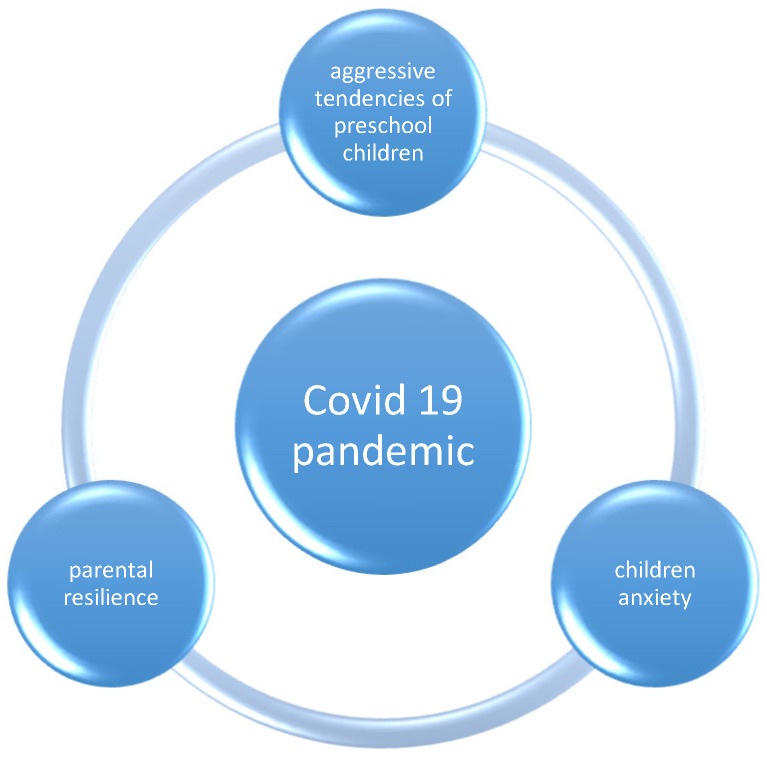
Relationship between parents’ psychological resilience levels and children’s aggression and anxiety levels in the COVID-19 pandemic.

In summary, this study shows that anxiety and parental resilience play a role in the aggressive tendencies of preschool children during the COVID-19 pandemic as seen in Figure 1. These results show that supporting development with more accurate approaches and interventions in early childhood, especially in crisis situations such as pandemics, is very important for child mental health.

## 5. Suggestions

This study examined the related role of anxiety and parental resilience on preschool children’s aggressive tendencies during the COVID-19 pandemic period. As the preschool period is developmentally important in terms of learning appropriate social behaviors and establishing psychological and behavioral patterns that will persist later in life, it can be said that studies conducted to determine the factors affecting children’s behavioral orientations will contribute significantly to the literature. This study is limited to 293 families and children. For this reason, it may be recommended to conduct similar studies with more sample groups in the preschool period. Also, this study is limited only to the Ankara province of Turkey. Finally, when the answers given by the families were evaluated, they may have given answers that they thought would make themselves and their children look better instead of honest answers, due to social desirability bias. It is very important for children’s mental health that future research aims to investigate what may affect children’s behavior during traumatic events such as natural disasters, epidemics, and earthquakes. Future studies can aim to develop intervention programs targeting the variables found to influence children’s behavioral orientations and evaluate the effectiveness of these programs. These intervention programs can also positively affect the classroom management of preschool teachers. In addition, educational interventions related to these factors could be organized for parents. In this way, families can have information about how they should behave in the face of variables that affect children’s behavioral tendencies.

## Figures and Tables

**Table 1:** Correlation results of aggression orientation scale and preschool anxiety scale.

		Social Anxiety	Generalized Anxiety	Separation Anxiety	Obvious Fears Anxiety	Total Preschool Anxiety
**Physical Aggression**	r *p*	0.11 0.06	0.19 0.01	0.19 0.01	0.13 0.02	0.21 0.01
**Relational** **Aggression**	r *p*	0.09 0.11	0.21 0.01	0.20 0.01	0.14 0.02	0.19 0.01
**Self-aggression**	r *p*	0.09 0.14	−0.04 0.49	0.05 0.36	−0.01 0.92	0.02 0.77
**Aggression towards objects**	r *p*	0.15 0.01	0.24 0.01	0.22 0.01	0.12 0.04	0.22 0.01
**Total Aggression**	r *p*	0.17 0.01	0.27 0.01	0.23 0.01	0.16 0.01	0.26 0.01

*p* < 0.05.

**Table 2:** Correlation results of brief resilience scale scores and aggression orientation scale and preschool anxiety scale total and subscale scores.

Brief Resilience Scale Scores		
	r	*p*
**Social Anxiety**	−0.26	0.01
**Generalized Anxiety**	−0.37	0.01
**Separation Anxiety**	−0.22	0.01
**Obvious Fears Anxiety**	−0.29	0.01
**Total Preschool Anxiety**	−0.38	0.01
**Physical Aggression**	−0.12	0.05
**Relational Aggression**	−0.11	0.06
**Self-aggression**	0.01	0.95
**Total Aggression**	−0.15	0.01

*p* < 0.05.

**Table 3:** Correlation results of aggression orientation scale scores with child age and number of children in family.

	Child’s Age		Number of Children	
	r	*p*	r	*p*
**Social Anxiety** **Generalized Anxiety** **Separation Anxiety**	0.11 0.12 −0.01	0.06 0.06 0.86	−0.05 −0.03 0.01	0.39 0.66 0.92
**Obvious Fears Anxiety** **Total Preschool Anxiety** **Psychological Resilience** **Physical Aggression** **Relational Aggression** **Self-aggression** **Aggression towards objects** **Total Aggression**	−0.02 0.06 0.05 −0.20 0.07 −0.06 −0.05 −0.12	0.69 0.29 0.38 0.01 0.24 0.34 0.36 0.05	−0.09 −0.06 0.02 0.12 0.05 −0.03 0.08 0.13	0.13 0.31 0.77 0.04 0.44 0.60 0.18 0.03

*p* < 0.05.

**Table 4:** Investigation of the effect of anxiety and parental resilience on children’s aggressive tendencies.

Dependent Variable: Aggression Orientation Scale Score							
Independent Variable	B	Std. Error	β	t	*p*	Durbin–Watson	VAF
**Fixed** **Preschool Anxiety Scale Scores** **R = 0.25** **Adjusted *R*^2^ = 0.06**	1.42 0.01 F = 19.78 *p* = 0.01	0.04 0.01	0.26	40.24 4.45	0.01 0.01	1.95	1
**Excluded Variable**	**B**	**t**	***p***				
**Strength Points**	−0.10	−1.62	0.106				

## Data Availability

The original contributions presented in the study are included in the article, further inquiries can be directed to the corresponding author.
